# A narrative review of AI monitoring in postoperative pain management and functional rehabilitation for spinal cord injury

**DOI:** 10.3389/fneur.2026.1838329

**Published:** 2026-07-09

**Authors:** Xiaoqing Zhang, Jianping Sheng

**Affiliations:** Department of Spinal Trauma, Shaoxing City Keqiao District TCM Hospital Medical Alliance General Hospital, Shaoxing, Zhejiang, China

**Keywords:** artificial intelligence monitoring, medical technology, pain management, rehabilitation therapy, spinal cord injury

## Abstract

Spinal cord injury (SCI) is a severe neurological condition often leading to significant disability, with causes including trauma, tumors, and spinal cord pathologies. Affected patients commonly present with limb motor dysfunction and persistent chronic pain. Current clinical management of SCI primarily relies on pharmacological and surgical interventions; however, neurological recovery remains limited due to the restricted neural regenerative capacity and the high costs associated with long-term rehabilitation, which together impose a substantial socioeconomic burden. AI monitoring, encompassing data acquisition and preprocessing, algorithmic modeling, real-time analysis, and decision support, offers the potential to continuously track vital signs, assess neurological function, and support pain management in patients with SCI through the integration of algorithms and real-time data analysis. Additionally, by facilitating the development of personalized rehabilitation plans, AI monitoring may enable more precise and dynamic postoperative management. Traditional post-SCI monitoring largely depends on subjective assessments and standardized interventions, which are often constrained by delayed responses and limited individual adaptation. As such, the integration of AI monitoring into the treatment process has attracted increasing attention from clinicians and researchers. This article aims to summarize the preliminary research progress and emerging applications of AI monitoring in postoperative pain management and functional rehabilitation for SCI, with the goal of providing a critical overview of its potential roles and current limitations in advancing post-SCI care.

## Graphical abstract

**Figure:**
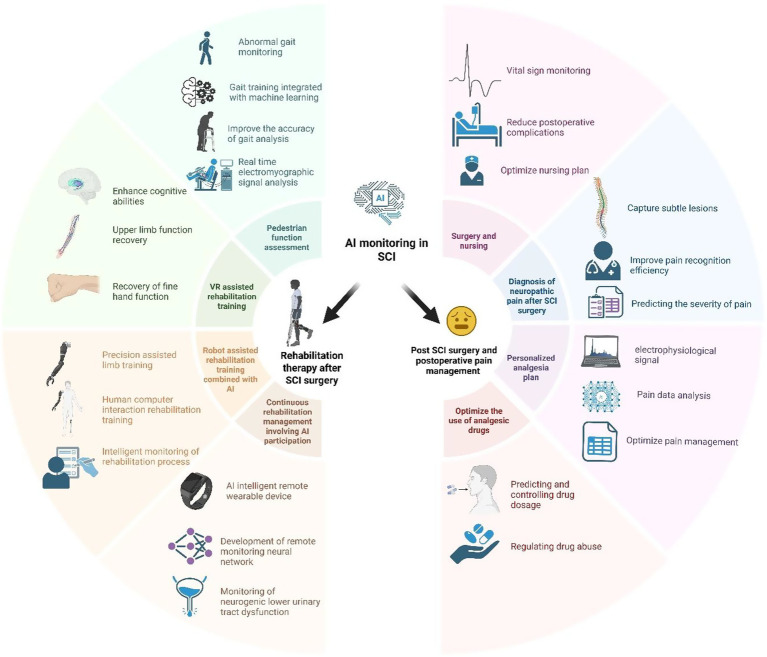

## 1 Introduction

Spinal Cord Injury (SCI) is a clinical syndrome characterized by damage to the structure or function of the spinal cord due to various causes, including trauma: vertebral fractures, intervertebral disc herniations; and disease: tumors, infections (epidural abscess, transverse myelitis), inflammatory conditions (multiple sclerosis), vascular disorders (spinal cord infarction), degenerative diseases (cervical spondylotic myelopathy), resulting in sensory, motor, reflex, and autonomic dysfunctions below the level of injury. It is a severe and permanent neurological condition that has a profound impact on the lives of patients. Epidemiological studies indicate that the global number of SCI cases is approximately 15.4 million (95% CI: 14,009,114–17,075,106), with approximately 5.6 million females (95% CI: 5,063,437–6,227,709). The incidence is higher in developed countries, whereas in developing countries, incidence has shown a progressive annual rise, largely attributable to an increasing burden of traumatic events (1). SCI can be classified into traumatic and nontraumatic types, with traumatic injury being the primary cause (2). However, the proportion of non-traumatic injuries has been rising in recent years, a trend largely driven by the increased prevalence of neurodegenerative diseases and malignant tumors (3).

A hallmark of SCI is a significant loss of spinal neurons at the lesion site. These injuries inhibit axonal regeneration and synaptic reorganization, ultimately leading to motor dysfunction in most patients. Owing to the challenges in neural regeneration, patients often experience lifelong motor impairments, which typically manifest as permanent paralysis or paresis below the level of injury, severe spasticity, muscle atrophy, and a loss of voluntary movement. These deficits frequently lead to a complete inability to walk, dependence on wheelchairs, and difficulty in performing essential activities of daily living independently, resulting in lasting physical disability (4). Severe loss of social function may contribute to cognitive deficits in patients with SCI and is associated with a significantly increased incidence of depression-related disorders. Studies have shown that 84.3% of patients with SCI exhibit depressive symptoms, with 60.8% experiencing a moderate-to-severe depressive mood (5, 6).

Following spinal cord injury, inflammatory responses and oxidative stress at the lesion site lead to neuronal death, resulting in a range of physical dysfunctions (7). Pain is a major post-SCI complication, with 53% of the patients reporting neuropathic pain, among whom 27% experienced pain below the level of injury and 19% reported pain at the level of injury (8). Neuropathic pain secondary to SCI is mediated by multiple mechanisms, including abnormal glial cell activity, ectopic discharge of nerve endings, and dysregulation of intracellular and extracellular signaling mechanisms (9). However, owing to the limited efficacy of conventional analgesic therapies, pain symptoms often remain refractory, further impairing patients’ daily functioning (10). The extremely low capacity for neural regeneration after SCI means that current rehabilitation strategies can only achieve partial functional recovery. Long-term rehabilitation resources are unevenly distributed, leading to insufficient home-based support in low-income regions and hindering progress toward social reintegration (11, 12). Given these challenges, postoperative pain management and rehabilitation are critical components of the recovery of patients with SCI. These represent not only essential extensions of clinical treatment but also constitute a crucial bridge enabling patients to transition from mere “survival” to “living” (13). Comprehensive post-SCI management provides multiple advantages. It helps prevent life-threatening complications, maximizes functional recovery, reduces healthcare costs, enhances social participation, and provides psychological and social adaptation support, thereby advancing progress toward precision medicine. However, due to disparities in social development and limited human resources, long-term, high-precision monitoring and management approaches have become a growing focus for both clinicians and patient families.

Advancements in artificial intelligence (AI) have enabled more efficient and comprehensive post-SCI monitoring. AI-based monitoring, with a technical core comprising data acquisition and preprocessing, algorithmic models, real-time analysis, and decision support, offers the potential for instantaneous patient supervision (14, 15). In conventional clinical practice, healthcare professionals often rely on traditional pain assessment scales and subjective patient reports to evaluate pain levels (e.g., VAS and NRS scores) (16). This approach is susceptible to inter-rater variability, potentially leading to substantial errors. Furthermore, traditional rehabilitation training often lacks the capacity for continuous, comprehensive data monitoring throughout the recovery process, resulting in missed detection of key indicators such as neuroelectrophysiological reflexes and electromyographic feedback (17).

Currently, the potential of AI monitoring in SCI rehabilitation is gradually being explored, with preliminary evidence supporting its transition from theoretical conceptualization to early-stage clinical investigation (18–21). AI monitoring may offer opportunities for more precise and timely disease prediction, as well as optimized treatment strategies, which could ultimately contribute to improved patient outcomes and reintegration into society. This article aims to critically review the current applications and future research directions of AI monitoring in spinal cord injury, with a particular focus on postoperative pain management and neurological functional recovery. By synthesizing existing preclinical and clinical evidence, this review seeks to address a gap in the literature by providing a comprehensive and critical overview of the current state of knowledge in this emerging field.

## 2 Methods

This is a narrative review based on literature retrieved from PubMed and Web of Science by using combinations of keywords, including but not limited to terms related to spinal cord injury (e.g., “spinal cord injury,” “SCI”), artificial intelligence (e.g., “artificial intelligence,” “machine learning,” “deep learning,” “AI”), and rehabilitation interventions (e.g., “pain management,” “rehabilitation,” “virtual reality,” “robotic-assisted training,” “remote monitoring,” “neurogenic bladder”). The search was supplemented by manual screening of reference lists of relevant articles. Articles were screened by title and abstract, with full-text review of potentially relevant papers. Data were extracted and synthesized narratively according to the main themes identified in the literature.

## 3 Core components of AI monitoring

Artificial Intelligence (AI) is a branch of computer science dedicated to creating systems capable of performing tasks that typically require human intelligence (22). Machine learning (ML) serves as the cornerstone of AI-based monitoring. The fundamental premise of ML is to enable computers to automatically learn patterns and regularities from data. Through extensive training on datasets, ML models learn underlying principles to subsequently generate predictions or decisions on new, unseen problems. In clinical decision-making, the principal algorithm types and their application characteristics of machine learning mainly include: supervised learning (including regression and classification, which commonly employs linear regression, support vector machines, decision trees, random forests and other ensemble methods, as well as neural networks and deep neural networks that achieve feature learning through multi-layer nonlinear transformations), unsupervised learning (which reveals hidden structures in data through methods such as principal component analysis and clustering), and reinforcement learning (which optimizes decision sequences through reward/punishment mechanisms) (23, 24).

Driven by algorithmic advances and technological innovations, machine learning has gradually evolved into deep learning, which is built upon architectures such as convolutional neural networks (CNNs), fully convolutional networks (FCNs), recurrent neural networks (RNNs), and generative adversarial networks (GANs). Deep learning can directly perform automatic learning and thereby enable computer-aided diagnosis and disease subtype classification. Moreover, integrating diverse data sources has shown promise in predicting disease prognosis and recommending appropriate postoperative treatment and care plans (25, 26).

In recent years, the integration of multimodal data has led to the increasingly widespread application of AI technologies in healthcare. Notable examples include medical image analysis (27), pathological diagnosis (28), novel drug discovery (29), and personalized patient health monitoring (30). Additionally, owing to its capability to accurately predict metrics, including healthcare costs, service utilization rates, and quality of care, AI is increasingly recognized by medical professionals as a valuable tool for healthcare management (31). Researchers have also combined AI models with portable devices such as smartphones and smartwatches to create simple and user-friendly monitoring tools. For instance, an AI-based analysis of dental selfies captured using smartphones has been shown to effectively monitor oral hygiene in the short term, thereby providing positive support for maintaining oral health (32).

In summary, AI and deep learning have shown considerable potential in identifying patterns within complex medical data, which may assist in diagnosis and personalized monitoring. These promising developments suggest potential applications in the specific and challenging domain of spinal cord injury detection.

## 4 Application of AI monitoring in post-SCI pain management

### 4.1 Pain prediction and dynamic monitoring

Pain is one of the most severe complications following spinal cord injury (SCI) surgery (33). Its incidence is influenced by various factors, including injury severity, type of surgery, postoperative care, and individual differences. It affects approximately 80% of patients, with a subset developing intractable pain (10, 34). Post-SCI pain can be categorized as nociceptive, neuropathic, or mixed-type pain (35). Nociceptive pain originates from musculoskeletal or visceral damage (e.g., neurogenic bowel) and can be managed with nonsteroidal anti-inflammatory drugs (NSAIDs) or local injections (36, 37). Neuropathic pain primarily manifests as central pain arising from abnormal neuronal excitability below the level of injury, or as peripheral pain resulting from intraoperative nerve root injury or postoperative scar compression (38). Conventional analgesics often have limited and frequently suboptimal efficacy against neuropathic pain (39). Mixed-type pain, with a complex etiology, currently lacks a definitive cure in clinical settings (35). Given the diverse and intricate pathogenic factors involved, determining how to manage post-SCI pain effectively has become a central challenge in clinical SCI care.

In recent years, AI technology has been increasingly integrated into clinical settings, with AI-based monitoring systems demonstrating potential to enhance diagnostic accuracy, treatment efficiency, and patient experience. By analyzing medical records and utilizing multimodal algorithms, AI offers potential advantages in SCI management by detecting subtle lesions that may be challenging for clinicians to identify, thereby enabling timely and comprehensive diagnosis (40, 41). One systematic study found that artificial intelligence achieved a mean AUC of 0.770 for prognostic prediction in traumatic spinal cord injury (mainly predicting AIS improvement, mortality, etc.), and a mean diagnostic accuracy of 0.898 (based on imaging modalities such as DTI, CT, and MRI) (42) (Table 1).

**Table 1:** AI applications in pain prediction and dynamic monitoring.

Disease/condition	AI/model type	Relevance to SCI	Sample size	References	Study design	Primary outcome/Target variable	Performance metrics	Comparator	Internal or external validation
Spinal cord injury (SCI)	Machine learning	SCI but non-postoperative	14 studies	(42)	Systematic review	The performance of AI algorithms in diagnosing and prognosticating tSCI	ACC = 0.898 (0.813–0.938); AUC = 0.770 (0.682–0.902)	The diagnostic and prognostic arms	NA
Spinal cord injury (SCI)	Deep learning	SCI but non-postoperative	NR	(53)	Preclinical research	Activation status of microglia after spinal cord injury	NA	Healthy group and SCI group	NA
Postoperative care (general)	Machine learning; deep learning	Non-SCI analogous evidence	NR	(46)	Systematic review	(1) Risk Identification, (2) Health Assessment, (3) Patient Classification, (4) Research Development, (5) Improved Care Delivery and Medical Records, and (6) Developing a Nursing Care Plan.	NR	NA	NA
Chronic pain (general)	Machine learning, data mining, and natural language processing	Non-SCI analogous evidence	30 studies	(48)	Scoping review	The actual outcomes of Al-based interventions for adult patients.	NA	NA	NA
Chronic pain (general)	Machine learning	Non-SCI analogous evidence	1,000 patients	(50)	Clinical cohort study	The time point was set to 6 months.	Sensitivity = 0.61905; Specificity = 0.65455; PPV = 0.14607; NPV = 0.94737; Balanced accuracy = 0.63680	Persistent pain group; non-persistent pain group	Internal validation

Furthermore, preliminary evidence suggests that AI-assisted monitoring may contribute to perioperative care. Studies indicate potential benefits in improving the success rate of spinal surgeries, reducing human error, optimizing perioperative workflows, and enhancing the quality of care. In the future, it may become possible to achieve continuous dynamic monitoring from the preoperative through the postoperative period by enabling risk stratification models to interoperate with intraoperative devices, such as control towers and anesthesia equipment. The system can automatically calibrate predictions based on real-time intraoperative parameters and recommend optimal postoperative monitoring strategies. This technological platform may integrate a variety of AI tools, including the hypotension prediction index, outcome prediction models, target-controlled infusion, closed-loop anesthesia, and early warning systems. In this environment, the core task of the clinician is to correctly interpret the information provided by these AI tools through clinical contextualization (43, 44). Postoperatively, AI systems enable real-time monitoring of patient vital signs and can promptly adjust nursing protocols, thereby facilitating the development of personalized care plans (45, 46) (Table 1). For instance, one study found that real-time vital sign monitoring technology incorporating AI was associated with a 10–30% reduction in the risk of serious postoperative complications during hospitalization (47). However, it should be noted that this finding is derived from a general surgical population and may not be directly generalizable to SCI patients without further validation.

In the realm of pain management, AI technology shows promise in improving the efficiency of pain identification and assessment, analyzes patient-reported pain data, predicts pain levels, and assists both clinicians and patients in managing chronic pain (48, 49) (Table 1). One study employed supervised machine learning to generate a brief questionnaire and found that it performed as effectively as the full-length questionnaire in predicting persistent postsurgical pain (50). Similarly, Nickerson et al. utilized neural network architectures to predict pain response and proposed that this novel approach yielded superior results to conventional methods (51). Moreover, by leveraging large datasets, AI can help identify complex disease patterns in SCI, potentially improving diagnostic accuracy, optimizing surgical procedures, and personalizing therapeutic interventions (52). Based on this, researchers developed a deep learning-based method to quantify microglial activation in preclinical spinal cord injury (SCI) models following treatment with drug-loaded nanovectors (52, 53). Personalized treatment strategies formulated through such approaches may hold promise for delivering more efficacious therapies to SCI patients (Table 1).

### 4.2 Development of personalized analgesia plans

AI monitoring may facilitate the comprehensive development of personalized analgesia plans by recording patient electrophysiological signals in real-time, dynamically analyzing pain data, and optimizing analgesic medication management. This approach has the potential to enhance the quality of care and improve patients’ quality of life (54). Evidence from pain research in other populations provides insights that may be relevant to SCI. A randomized clinical trial on pain demonstrated that while the therapeutic efficacy of AI-guided and interactive voice response-based personalized cognitive behavioral therapy for chronic pain (AI-CBT-CP) showed no significant difference compared with standard telephone-delivered CBT-CP at 3 and 6 months, the AI-CBT-CP intervention improved their functional levels, improved their cognition of pain intensity, and reduced the intervention time required by therapists (55) (Table 2). Although this study focused on general chronic pain rather than SCI specifically, its findings suggest that AI-assisted psychological interventions warrant investigation in SCI populations.

**Table 2:** AI applications in personalized analgesia plan development.

Disease/condition	AI/model type	Relevance to SCI	Sample size	References	Study design	Primary outcome/Target variable	Performance metrics	Comparator	Internal or external validation
Spinal degenerative diseases	“Machine Learning,” “Deep Learning,” “Neural Network,” “Computer Aided Diagnosis”	Non-SCI analogous evidence	57 studies	(66)	Systematic review	The available literature on the use of CAD in the diagnosis and treatment of chronic LBP	Used machine learning (median accuracy = 92.5%); used deep learning (median accuracy = 88.8%)	NA	NA
Spinal conditions (pain quantification)	machine learning model	Non-SCI analogous evidence	60 patients	(67)	Clinical research	Pain level feedback	ACC = 71%; positive predictive value = 0.71; the overall F1 score was 0.73.	NR	Internal validation
Chronic pain (general)	AI-guided personalized cognitive behavioral therapy (AI-CBT-CP), interactive voice response	Non-SCI analogous evidence	278 patients	(55)	Randomized controlled trial	The primary outcome was the Roland Morris Disability Questionnaire (RMDQ; range 0–24), measured at 3 months (primary end point) and 6 months.	A greater proportion of patients receiving AI-CBT-CP had clinically meaningful improvements at 6 months as indicated by RMDQ (37% vs. 19%; *p* = 0.01) and pain intensity scores (29% vs. 17%; *p* = 0.03).	CBT-CP group; AI-CBT-CP group	NA
General pharmacotherapy (vancomycin dosing)	Machine learning	Non-SCI analogous evidence	2,282 patients	(57)	Cohort study	Vancomycin dosage titration	PAR of initial dose model was 51.7%, and subsequent dose model was 73.4%	Training group, validation group, and test group.	Internal validation
Idiopathic membranous nephropathy (Chinese and Indian patients)	Machine learning	Non-SCI analogous evidence	463 patients	(58)	Clinical research	Proteinuria change rates from baseline	Emax = −72.7%	NA	Internal validation
Low back pain (workers)	AI-based health promotion system	Non-SCI analogous evidence	94 patients	(62)	Randomized blind study	The subjective severity of the neck and shoulder pain/stiffness and low back pain after 12 weeks	NR	Intervention group (Artificial Intelligence Assisted) and control group	NA
Low back pain (general population)	Portable mobile device-based AI-personalized service app	Non-SCI analogous evidence	461 patients	(63)	Randomized controlled study	Global perceived effect (GPE)/pain self-efficacy (PSEQ)/satisfaction/app engagement.	NR	usual care group; usual care plus selfBACK group	NA
Pain assessment	Machine learning	Non-SCI analogous evidence	89 studies	(65)	Systematic review	Machine learning methods for assessing pain using physiological signals	NR	NR	NA
Experimental/postoperative/long-term pain (general)	electrocardiographic-derived measures’ ability	Non-SCI analogous evidence	15 studies	(64)	Systematic review	Pain thresholds assessment	NR	NR	NA
Breast cancer surgery	Machine learning	Non-SCI analogous evidence	1,000 patients	(61)	Clinical research	Identify parameters that predict persistence of significant pain	Cross-validated accuracy = 86%; negative predictive value = 95%	persisting pain group and non-persisting pain groups	Internal validation

Personalized dosage recommendation systems using AI can collect large-scale datasets to dynamically predict and control drug dosage utilization, thereby improving therapeutic efficacy and preventing adverse drug reactions (56) (Table 2). While direct evidence in SCI pain management is limited, studies in other clinical contexts demonstrate the potential of this approach. In a study by Wang Z, a machine learning model was developed to assist in vancomycin dose adjustment, and the results indicated that the machine learning model outperformed traditional models in pharmacokinetic indices, specifically in Prediction Error Rate (PER) and Mean Absolute Error (MAE) (57) (Table 2). Furthermore, researchers have utilized machine learning to optimize tacrolimus dosage in Chinese and Indian patients with idiopathic membranous nephropathy, achieving superior treatment outcomes (58) (Table 2). These examples illustrate the broader applicability of AI-driven dosage optimization, which could potentially be adapted for analgesic management in SCI, though direct studies are needed.

Regarding the use of analgesics, the overuse of opioids and the associated deaths in some countries have become serious public health crises (59). Researchers have developed AI-based software prototypes capable of identifying safety signals for opioid-related adverse events from free text in electronic health records and discharge summaries. This software enhances the efficiency of opioid safety monitoring and alleviates the burden of manual research (60). Given the challenges of opioid management in SCI, this application may offer a useful monitoring strategy; however, its feasibility and performance in the postoperative SCI setting have not been directly studied.

Simultaneously, the advancement of AI technology is contributing to improved prediction of clinical pain prognosis, although most studies to date have focused on non-SCI populations. Lorch et al. collected data from 1,000 patients treated for breast cancer and utilized supervised learning to identify 21 parameters for constructing a classifier for predicting persistent postoperative pain. This classifier achieved a prediction accuracy of 86% and negative predictive value of approximately 95%, effectively aiding the clinical prediction of post-breast cancer surgery pain (61) (Table 2). While this population differs from SCI patients, the methodological approach—using machine learning to integrate multiple clinical parameters for pain prediction—could inform future research in SCI. Tomomi et al. discovered that an AI-based health promotion system significantly improved low back pain symptoms in workers (62) (Table 2). Concurrently, a randomized controlled trial involving 461 participants demonstrated that a portable mobile device-based AI-personalized service app effectively alleviated LBP symptoms in users over nine months (63) (Table 2). These findings in low back pain populations suggest that similar approaches might be explored for SCI-related pain, though the underlying pain mechanisms differ substantially. Wegeberg et al. found that electrocardiogram-derived autonomic data can predict pain development, revealing that high levels of parasympathetic activity effectively predict experimental, postoperative, and long-term pain (64) (Table 2). This physiological approach may have more applicability to SCI, as autonomic dysfunction is common in this population.

Although accurate assessment of pain still faces significant challenges in clinical practice, AI can diagnose underlying pain by analyzing pain indices, previous pain history, electromyography data, and other human pain biomarker (65) (Table 2). Furthermore, high-level AI-assisted systems have achieved an average diagnostic accuracy exceeding 80% for spinal degenerative diseases (66) (Table 2). These applications may relevant to SCI care, as many SCI patients have concomitant spinal pathology. Additionally, Akiro et al. utilized digital phenotyping, incorporating smartphone voice data and self-reported pain surveys, to refine pain quantification in spinal conditions and predict daily pain levels (67) (Table 2).

Taken together, this evidence suggests that AI holds promise for predicting the efficacy and risks of different drugs and dosages based on patient characteristics. While direct evidence in SCI populations remains limited, the approaches developed in other clinical contexts—including dosage optimization, pain prediction modeling, and digital phenotyping—provide a foundation for future research investigating personalized analgesic regimens for individuals with SCI. Such research would need to validate these approaches specifically in SCI populations and address the unique pain mechanisms and clinical considerations associated with spinal cord injury.

## 5 The role of AI in functional rehabilitation

### 5.1 Walking function assessment

Rehabilitation therapy is crucial for the functional recovery of patients with spinal cord injury (SCI). Owing to the challenges associated with neurological recovery post-SCI surgery and traditional rehabilitation methods that rely heavily on manual assistance and subjective functional assessments, which often fail to objectively and accurately capture patients’ physical data, a growing number of researchers are focusing on AI technologies. The integration of AI with neurological rehabilitation represents an emerging area of investigation with potential applications for patients with neurological impairments, including those with SCI (68, 69).

Motor function recovery, particularly lower-limb walking ability, is the key to physical recovery in patients with SCI during the postoperative period. This is central to their reintegration into society and serves as a significant predictor of postoperative complications and mortality. A clinical study indicates that, in rehabilitation assessment, the application of machine learning can significantly improve the post-discharge evaluation of activities of daily living (ADL) function and the determination of rehabilitation exercise effectiveness in postoperative SCI patients (70) (Table 3). This evidence suggests that the application of artificial intelligence has potential positive implications for the functional recovery of SCI patients after surgery. In gait analysis, studies have found that, compared to traditional statistical analysis, machine learning, by utilizing candidate gait parameters for feature selection and model construction, can predict and distinguish different walking phases, as well as identify abnormal gaits and their severity (71) (Table 3). Hwang et al. developed an inertial measurement unit (IMU)-based algorithm for classifying abnormal gaits and reported recognition accuracy of 91% (72) (Table 3). Another intelligent gait recognition method (IGRM) based on machine learning achieved recognition accuracies of 92.41 and 97.79% for the static and dynamic components of a pathological gait, respectively, in the same child, and recognition rates of 85.78 and 78.81% for pathological gaits across different children (73) (Table 3). While these findings demonstrate the technical feasibility of AI-based gait analysis, it should be noted that these studies were conducted in general populations with gait abnormalities rather than specifically in SCI patients. The transferability of these algorithms to SCI-specific gait patterns requires further validation.

**Table 3:** AI applications in walking function assessment and gait analysis.

Disease/condition	Technology used	Relevance to SCI	Sample size	References	Study design	Primary outcome/Target variable	Performance metrics	Comparator	Internal or external validation
Spinal cord injury (SCI)	Machine learning	Postoperative SCI	1231patients	(70)	Clinical research	The rehabilitation effect of patients after rehabilitation training.	RF model: MAE = 0.0875, RMSE = 0.1346, R^2^ = 0.7662 HHO-RF model: MAE = 0.0821, RMSE = 0.1089, R^2^ = 0.8537	NA	Internal validation
Gait abnormalities (general population)	Machine learning	Non-SCI analogous evidence	30 patients and 50 healthy people	(71)	Clinical research	Target stepping, slalom walking, obstacle avoidance, and speed adaptation.	ACC = 0.82, SEN = 0.8, F1-score = 0.77, ROC-AUC = 0.75	NA	Internal validation
Gait abnormalities (general population)	Machine Learning	Non-SCI analogous evidence	10 healthy people	(72)	Clinical research	Gait analysis	ACC = 91%	NA	Internal validation
Pathological gait (pediatric population)	Machine Learning	Non-SCI analogous evidence	17 children	(73)	Clinical research	Pathological gait recognition	IGRM: intra-subject: ACC = 92.41% (static); ACC = 97.79% (dynamic) IGRM: inter-subject: ACC = 85.78% (static); ACC 78.81% (dynamic)	NA	Internal validation
Healthy adults	Pose estimation AI (OpenPose)	Non-SCI analogous evidence	21 healthy people	(75)	Clinical research	Gait analysis data	Camera side: CMC = 0.936–0.994; opposite side: CMC = 0.890–0.988	NA	Other (concurrent validity)
Diverse patient populations	Multimodal sensors with AI algorithms for gait analysis	Non-SCI analogous evidence	66 studies	(76)	Systematic review	Gait analysis	NR	NA	NA
Neuromotor disorders (various, undergoing robot-assisted gait rehabilitation)	Artificial intelligence (AI) algorithms	Non-SCI analogous evidence	46 patients	(74)	Clinical research	Predict the level of patient engagement during RAGR.	Support Vector Machine model: F1 score = 95.6%; Extreme Gradient Boosting model: F1 score = 95.4%	NA	Internal validation

Furthermore, Simone et al. investigated the application of five artificial intelligence algorithms to structured heart rate variability and electrodermal activity features to comprehensively predict engagement in patients with neuromotor disorders during robot-assisted gait rehabilitation (74) (Table 3). This study included patients with various neuromotor disorders, and although SCI was not the sole focus, the methodological approach may inform future research in SCI rehabilitation. Similarly, another team validated the effectiveness of an AI-based single-camera gait analysis system for measuring bilateral lower limb kinematics. The results showed a Mean Absolute Error (MAE) of 2.3° to 3.1° on the camera side and 3.1° to 4.1° on the contralateral side, whereas the multiple correlation coefficients ranged from 0.936 to 0.994 on the camera side and 0.890 to 0.988 on the contralateral side. These findings indicate that single-camera gait analysis is sufficiently accurate for clinical assessment (75) (Table 3). However, this validation was performed in healthy adults rather than SCI patients, and further studies are needed to establish its accuracy in SCI populations with varying levels of impairment. To evaluate the application of AI in gait analysis in greater detail, a systematic review of 66 studies found that most studies used multiple sensors to record and analyze the gait. Among these, 40 studies did not employ AI and analyzed only kinematic and kinetic gait parameters, whereas 26 studies integrated multimodal sensors with AI algorithms for analysis. A systematic review concluded that combining multimodal data with AI improves the quality and precision of gait analysis (76) (Table 3). This systematic review provides a comprehensive overview of the field, but it is important to note that the included studies covered diverse patient populations, and the specific benefits for SCI gait analysis remain to be fully characterized.

In summary, emerging evidence suggests that integrating AI into gait analysis may help address some limitations of traditional methods, including subjectivity and measurement error. The development of miniaturized electronic components, increased computational power, and advances in neural networks may enable future integration of implantable miniature monitoring sensors with AI for rehabilitation assessment. Continuous electromyography signals, joint range-of-motion assessments, and muscle strength evaluations could provide clinicians with more detailed physiological data, potentially enabling more precise and appropriate treatment regimens. However, most current evidence derives from non-SCI populations, and direct validation in SCI patients is needed before clinical implementation.

### 5.2 Intelligent rehabilitation intervention

Virtual Reality (VR), as a potentially effective intervention system, has been investigated for its role in promoting functional recovery. VR-based interventions aim to facilitate recovery by creating versatile and immersive environments and facilitating personalized interactions (77). A meta-analysis of VR studies published between 2003 and April 2023, involving 130 participants across three studies, found that VR shows efficacy for cognitive rehabilitation in older adults (≥65 years) with mild cognitive impairment (MCI). The results indicated that VR-based rehabilitation therapy was at least as effective as conventional cognitive rehabilitation therapy in assessing the overall cognitive function, executive function, and functional status, suggesting the potential advantages of VR–CRT in MCI treatment (78) (Table 4). While this evidence supports VR efficacy in MCI, its direct applicability to SCI rehabilitation requires investigation, as the mechanisms of cognitive impairment and rehabilitation needs differ between these populations.

**Table 4:** AI and related technologies in intelligent rehabilitation intervention.

Disease/Condition	Technology used	Relevance to SCI	Sample size	References	Study design	Primary outcome/Target variable	Performance metrics	Comparator	Internal or external validation
Spinal cord injury (SCI)	Robot-assisted locomotor training (activity-based therapy)	SCI but non-postoperative	30 patients	(86)	Randomized controlled study	The voluntary movement was qualified by measuring active range of motion, maximal velocity peak and trajectory smoothness for the spastic ankle during a movement from full plantar-flexion (PF) to full dorsi-flexion (DF) at the patient’s maximum speed. Dorsi- and plantar-flexor muscle strength was quantified by isometric maximal voluntary contraction (MVC).	NR	Experimental group (receiving robot training); Control group (not receiving robot training)	NA
Spinal cord injury (SCI)	Telerehabilitation	SCI but non-postoperative	6 people	(94)	Sampling analysis	To explore urban occupational therapists’ perspectives on the benefits of and barriers to telerehabilitation use with SCI.	NR	NA	NA
Spinal cord injury (SCI)	Telerehabilitation	SCI but non-postoperative	30patients	(95)	Double blind randomized controlled trial	The Spinal Cord Independence Measure (SCIM III) (primary outcome measure) and Coronavirus Anxiety Scale (CAS) (secondary outcome measure) were evaluated at baseline, four weeks, and eight weeks.	NR	Telerehabilitation or control group	NA
Spinal cord injury (SCI) with ULUTD	Machine learning	SCI but non-postoperative	1,263 patients	(97)	Clinical research	The total Neurogenic Bladder Symptom Score (NBSS) and the NBSS-satisfaction question (QOL).	The QOL score for the high function, low SCI complication group had more improvement (*β* = −0.12, *p* = 0.005), while the female predominant group had more deterioration (β = 0.09, *p* = 0.047).	“Female predominant,” “high function, low SCI complication,” “quadriplegia with bowel/bladder morbidity,” and “older, high SCI complication.”	Other (clinical criterion validity; consensus clustering)
Spinal cord injury (SCI) with urinary tract infection	Prediction models	SCI but non-postoperative	558 patients	(98)	Clinical research	Analyze the influencing factors of UTI in hospitalized patients with spinal cord injuries.	Training group: AUC = 0.808 (95%CI = 0.760–0.856); verification group: AUC = 0.767 (95%CI = 0.688–0.845)	Training group (*n* = 390) and verification group (*n* = 168)	NR
ULUTD	Machine learning	SCI but non-postoperative (involve)	18 studies	(99)	Systematic review	Studies applying AI to uroflowmetry, cystometry, or pressure-flow studies in human participants were included.	Raw signal-based models AUC above0.85, exceeding 0.90	NA	NA
Neurogenic lower urinary tract dysfunction (ULUTD)	Machine learning; deep learning	Non-SCI analogous evidence	NR	(96)	Review	NA	NA	NA	NA
People without a history of lower limb diseases	Brain-Controlled Hip Exoskeleton	Non-SCI analogous evidence	40 people	(88)	Preclinical research	gait control	NA	NA	Internal validation (algorithm section)
Mild cognitive impairment (MCI) in older adults (≥65 years)	VR-based cognitive rehabilitation therapy	Non-SCI analogous evidence	3 studies	(78)	Systematic review	the efficacy and effectiveness of VR-based cognitive rehabilitation therapy	NR	NA	NA
Chronic stroke	Mixed Reality (MR)-based upper limb rehabilitation intervention	Non-SCI analogous evidence	30 patients	(84)	Reverse design research	Reverse Design Research; Reverse Design Research; Manual Function Test, Wolf Motor Function Test, Box and Blocks Test, Nine Hole Peg Test; Motor Activity Log; System Usability Scale, Intrinsic Motivation Inventory	NR	Single group self comparison	NA
Various neurological conditions (systematic review of 12 RCTs)	Fully immersive virtual reality (FIVR)	Non-SCI analogous evidence	12 studies	(85)	Systematic review	investigate the efficacy of FIVR in stroke rehabilitation	NR	NA	NA
Unilateral limb motor dysfunction (various etiologies)	Gait rehabilitation robots	Non-SCI analogous evidence	20 people; 6 patients	(89)	Clinical research	Accurate gait event detection	NR	Control group and experimental group (with differences in rehabilitation methods)	NA
Various patient populations	Remote rehabilitation monitoring	Non-SCI analogous evidence	15 studies	(90)	Systematic review	Report the monitoring devices currently proposed and tested for shoulder rehabilitation in home settings.	NR	NA	NA
Neurological conditions (general)	Deep learning	Non-SCI analogous evidence	93 patients; 73 healthy people	(92)	Dataset research	Predict exact symptom value of Parkinson’s Disease patients	Achieving = 99.5%; ACC = 98.7%; Sensitivity = 99.1%	93 Parkinson’s disease patients and 73 control group participants	Internal validation
Stork	The random forest and extremely randomized trees algorithm	Non-SCI analogous evidence	120 patients	(93)	Clinical research	Assess the Fugl-Meyer upper extremity subscale for post-stroke patients with motor dysfunction	ACC = 92.66%	NA	Internal validation
Various neurological impairments	AI-powered electromyography-driven robotic hand for upper limb functional recovery	Non-SCI analogous evidence	20 patients	(87)	Randomized controlled study	The Fugl-Meyer assessment of UE motor function (FMA), motor activity log-14 amount of use score (MAL-14 AOU), modified Ashworth scale (MAS), H reflex, and reciprocal inhibition	NR	Active group; control group	NA

VR technologies range from fully immersive systems, which isolate patients in a virtual environment for motor and cognitive rehabilitation, to non-fully immersive systems, which use standard screens for early-stage balance and coordination exercises (79, 80). Primarily utilizing Augmented Reality (AR) and Mixed Reality (MR) technologies to overlay digital cues onto the real world, VR helps patients perform recovery exercises such as reaching or grasping (81–83). Evidence for MR-based interventions comes primarily from stroke rehabilitation. A study investigating the clinical effectiveness of an MR-based intervention on upper limb function in 30 patients with chronic stroke found that after 30 sessions of conventional physical therapy and 30 sessions of experimental system training, the patients showed significant improvements in arm function (Wolf Motor Function Test, *p* < 0.01), finger dexterity (Box and Blocks Test, *p* < 0.01; Nine Hole Peg Test, *p* < 0.01), and engagement (*p* < 0.01), which were maintained until the end of the study. The system has been reported to be highly usable, enjoyable, and motivating, supporting the application of MR interventions for upper limb rehabilitation in patients with chronic stroke (84) (Table 4). Similarly, a systematic review of 12 randomized controlled trials (234 males and 115 females) found that VR applications, compared with traditional rehabilitation, led to significant improvements in upper limb dexterity, gait performance, and dynamic balance, thereby enhancing patient independence and quality of life (85) (Table 4). However, these reviews included studies across various neurological conditions, and the specific evidence for SCI remains limited.

In the context of SCI rehabilitation, robotic-assisted technologies have been investigated as a complementary approach to traditional therapy. Varoqui confirmed that activity-based therapy (ABT), such as robot-assisted locomotor training, can improve walking speed and distance in SCI, effectively restoring limb function (86) (Table 4). These studies provide direct evidence in SCI populations, supporting the potential of robot-assisted interventions. Furthermore, a study on the value of an integrated AI-powered electromyography-driven robotic hand for upper limb functional recovery in patients with neurological impairments showed that compared with traditional rehabilitation models, patients using the integrated AI robotic hand demonstrated significant improvements in the Fugl-Meyer Assessment (FMA), Amount of Use score from the Motor Activity Log-14 (MAL-14 AOU), and Modified Ashworth Scale (MAS), with effects persisting after 4 weeks (87) (Table 4). While this study included patients with various neurological impairments, the findings suggest that AI-integrated rehabilitation robots may have applications in SCI upper limb rehabilitation. A research team designed a self-aligning, hybrid-controlled hip exoskeleton by integrating brain-computer interface with augmented reality visual stimulation. Experiments showed that the subjects achieved a reduction in task time by 18.64%, a decrease in instantaneous rate by 28.31%, and an increase in output ratio by 7.36%, demonstrating superior gait rehabilitation outcomes compared to traditional models (88) (Table 4). This proof-of-concept study, conducted in individuals without spinal cord injury (SCI), does not constitute evidence for the feasibility of brain-computer interface-controlled exoskeletons in patients with SCI; while the findings are promising, dedicated investigations in the SCI population are required to explore any potential application. Correspondingly, a study on an AI-integrated rehabilitation training system found that through algorithm optimization and model construction, an intelligent robot significantly reduced the gait assessment time for patients with unilateral limb motor dysfunction and served as an effective tool for monitoring patient rehabilitation progress (89) (Table 4). This study’s population included patients with various etiologies of unilateral dysfunction, limiting conclusions specific to SCI.

Remote rehabilitation monitoring represents another application of AI in SCI care. By lightly deploying the core AI algorithms used in rehabilitation robots, such as motion analysis and functional assessment, on wearable sensors and mobile terminals, remote, objective, and real-time monitoring of patient home-based rehabilitation training may be achieved. This approach could potentially enable therapists to observe patient performance at home and, through AI data analysis, facilitate dynamic adjustment of rehabilitation plans. Evidence from other populations supports the potential of this approach. Remote rehabilitation monitoring has been reported to be effective in improving activities of daily living (ADL), balance, health-related quality of life, and depressive symptoms in various patient populations (90, 91) (Table 4). Currently, one research team has applied deep learning technology and regression algorithms to wearable devices for neurological systems. Combined with deep learning, the device achieved a disease detection and classification accuracy of 99.5%, with a sensitivity of 98.7% and a specificity of 99.1%, significantly outperforming most existing studies (92) (Table 4). However, this study’s methodology and validation population require careful examination before extrapolation to SCI. A study on artificial intelligence–based assessment of upper limb and hand function in post-stroke patients found that the AI model achieved a scoring accuracy of 92.66% on the Fugl-Meyer items (93). This stroke-specific tool demonstrates the feasibility of AI-based remote monitoring for neurological rehabilitation, suggesting similar approaches could be developed for SCI. Furthermore, questionnaire surveys for patients with SCI indicate that addressing challenges in remote rehabilitation through digital technology provides significant support for functional recovery (94, 95) (Table 4). These patient-reported outcomes highlight the perceived value of remote rehabilitation among individuals with SCI.

Emerging AI-based monitoring approaches are also being explored for neurogenic lower urinary tract dysfunction (NLUTD), a major determinant of morbidity and quality of life after SCI. Machine learning algorithms applied to urodynamic, clinical, and neuroimaging data have demonstrated strong potential to identify patients at risk of therapeutic nonresponse and enable individualized management, marking a shift toward data-driven precision medicine in neurourology (96) (Table 4). In the context of SCI, unsupervised machine learning has been successfully used to identify distinct bladder-relevant phenotypes from 1,263 patients, facilitating cluster-based targeted strategies to enhance bladder-related outcomes and quality of life (97) (Table 4). Furthermore, prediction models for urinary tract infections—a frequent complication of NLUTD—have been constructed and verified specifically for hospitalized SCI rehabilitation patients (98) (Table 4). A systematic review of AI in urodynamic reported AUC values exceeding 0.90. Machine learning models accurately identified detrusor overactivity, underactivity, and bladder outlet obstruction, supporting automated urodynamic interpretation (99) (Table 4). These advances suggest that AI-based monitoring could extend beyond pain management and motor rehabilitation to address NLUTD, a critical yet often undermanaged domain in SCI care. However, most studies remain limited by small sample sizes and lack of external validation, underscoring the need for large-scale, multicenter validation in SCI-specific populations before clinical implementation.

In conclusion, AI-assisted rehabilitation technologies, including intelligent gait analysis, VR/AR-based interventions, robotic-assisted training, and remote monitoring systems, show promise for enhancing functional recovery in patients with neurological impairments. Emerging evidence also suggests potential for AI-based monitoring in managing neurogenic lower urinary tract dysfunction, a key determinant of quality of life after SCI. For SCI, direct evidence exists exclusively for robotic-assisted locomotor training; evidence for brain-computer interface applications and other modalities derives largely from non-SCI populations. AI may contribute to more precise measurement and analysis, generation of objective data reports, and identification of imperceptible movement details. However, the current evidence base requires further validation through well-designed studies specifically in SCI populations. Future research should focus on developing and validating AI-based rehabilitation approaches tailored to the unique needs and characteristics of individuals with spinal cord injury, with clearly defined outcomes and standardized assessment protocols.

## 6 AI monitoring in clinical medical practice

The rapid advancement of artificial intelligence enabled its application across various stages of medical practice, with emerging evidence suggesting potential benefits in multiple clinical settings. In the prehospital phase, intelligent voice recognition software has been shown to significantly improve the recognition efficiency of stroke patients and increase the success rate of thrombolytic therapy (100). Regarding emergency triage, the intelligent risk-scoring system developed by Chen et al. can effectively stratify the risk factors for in-hospital cardiac arrest, providing substantial assistance to clinicians in making accurate decisions (101). Furthermore, evaluations of severe sepsis prediction algorithms have indicated that by screening high-risk factors, AI prediction models can effectively reduce in-hospital sepsis mortality (102). These applications demonstrate AI’s capacity to enhance the work efficiency and accuracy of healthcare professionals in specific clinical contexts. However, it is important to note that these findings are derived from studies in stroke, cardiac arrest, and sepsis populations, and their direct applicability to spinal cord injury (SCI) care requires separate investigation.

Specific to spinal cord injury, Fan et al. developed a machine learning (ML) prediction model that effectively forecasts prolonged length of stay in both the intensive care unit (ICU) and hospital for SCI patients. This study provides direct evidence in SCI populations, demonstrating that AI-based prediction models can assist clinicians in managing bed turnover and planning care for individuals with SCI (103).

In summary, AI technology has demonstrated potential for enhancing disease recognition, risk stratification, and prognosis prediction across various medical domains. For SCI specifically, current direct evidence includes applications in predicting length of stay, while evidence for other AI applications in SCI clinical practice remains limited. The integration of AI into clinical workflows may improve operational efficiency and accuracy of healthcare delivery. However, the extent to which findings from non-SCI populations can be extrapolated to SCI care requires careful consideration and validation through studies specifically designed for SCI populations. Future research should focus on developing and validating AI-based clinical tools tailored to the unique needs and characteristics of individuals with spinal cord injury, with clearly defined outcomes and standardized assessment protocols.

## 7 Challenges in AI monitoring

The prospects for AI monitoring in the medical field are undoubtedly promising; however, several significant shortcomings persist. The primary challenges were patient privacy and security. Healthcare systems provide vast amounts of patient data to AI servers for algorithmic training without patient consent. The unauthorized use of patient data in training algorithms constitutes a profound disrespect for medical privacy. The second major challenge is the “black-box” nature and lack of interpretability. Current research applying AI technology to the treatment and rehabilitation of conditions such as SCI often suffers from small sample sizes and insufficient evidence to substantiate its feasibility. If problems arise, this could lead to strained doctor-patient relationships, diminished treatment adherence, and eroded trust. The third challenge involves potential societal issues. With advances in computer science, the accuracy of AI treatments has improved markedly. This progress has raised concerns among healthcare professionals about the possibility that AI could become obsolete in the future.

## 8 Conclusion

In conclusion, AI-based monitoring represents an emerging tool in post-SCI care, with demonstrated potential in several areas including pain management and neurological rehabilitation. Based on the evidence reviewed in this article, the following applications show promise: (1) Data integration for pain assessment: Collecting diverse parametric data may facilitate the establishment of pain assessment models, enabling pain quantification, prediction, and dynamic monitoring. However, direct evidence in SCI populations remains limited, and most pain prediction models have been developed in non-SCI populations. (2) Personalized analgesia monitoring: Through the collection of metrics such as ECG-derived data, this approach may enable real-time tracking of patient pain status and timely adjustment of medication types and dosages, potentially facilitating personalized analgesia regimens. Preliminary evidence from general chronic pain populations supports this approach, but validation in SCI-specific pain management is needed. (3) Rehabilitation assessment: AI may assist in grading neurological injury based on imaging and electrophysiological signals, evaluating rehabilitation progress, and providing prognostic predictions. Direct evidence in SCI exists for robotic-assisted locomotor training, while AI-based gait analysis and functional assessment tools require further validation in SCI populations. (4) Technology-assisted rehabilitation training: By integrating virtual reality with AI-based monitoring, diverse therapeutic scenarios can be constructed to deliver personalized rehabilitation exercises. Assisted rehabilitation devices such as AI-integrated robotic hands may enable more comprehensive assessment of recovery capacity and support standardized training protocols. Evidence for VR/AR-based interventions derives primarily from stroke and other neurological populations, with limited direct validation in SCI. (5) Remote monitoring capabilities: AI-supported systems for remote monitoring and home-based rehabilitation may enable training anytime and anywhere. Patient-reported outcomes suggest that individuals with SCI value remote rehabilitation approaches, though objective evidence of efficacy in this population requires further investigation. In addition, AI-enhanced remote monitoring has shown early promise for managing neurogenic lower urinary tract dysfunction (e.g., wearable bladder sensors, machine learning analysis of urodynamic data), which could address a major quality-of-life determinant after SCI, but requires dedicated validation in this population.

Despite its numerous benefits, AI monitoring has apparent drawbacks. First, the data-related issues are of paramount importance. Lack of data diversity and potential data latency can lead to a series of missed or misdiagnosed cases. The question of liability attribution in cases where medical errors occur due to AI-assisted diagnostics remains unresolved. Concurrently, data breaches can exacerbate doctor-patient conflict. Second, current AI monitoring technology has inherent limitations: algorithms often rely heavily on historical data, making real-time dynamic clinical decision-making difficult, and the “black-box” nature of the decision-making process results in insufficient explainability, easily raising doubts about the reliability of the results among both patients and healthcare providers. Furthermore, the widespread application of AI may introduce ethical and social challenges, including exacerbating disparities in healthcare resource distribution, diminishing humanistic care in doctor-patient relationships, and raising concerns among medical staff about job displacement.

To address these challenges, future efforts should focus on: (1) integrating multimodal medical data and promoting data resource sharing to build high-quality, standardized clinically applicable databases; (2) developing AI systems with visualizable decision pathways and natural-language explanation capabilities to foster explainable and trustworthy medical AI applications; (3) conducting well-designed studies specifically in SCI populations to validate AI-based approaches; and (4) establishing clear regulatory frameworks for liability attribution and data protection.

Based on the current evidence, we propose that post-SCI care may benefit from AI monitoring in several areas, including complication prevention, functional recovery assessment, and pain management. However, these potential benefits must be considered alongside the current limitations in the evidence base. Interdisciplinary collaboration among clinical staff, physicians, software engineers, and medical device developers is necessary to improve the maturity of AI applications in SCI care. Integrating multiomics dynamic monitoring with AI predictive models represents a promising direction for future research, potentially enabling personalized approaches spanning risk intervention to functional reconstruction. Low-cost wearable devices and intelligent learning technologies have the potential to overcome resource barriers, potentially enabling access to rehabilitation services in remote areas. However, these technologies require rigorous evaluation in diverse settings before widespread implementation.

In summary, AI monitoring shows promise for enhancing pain management and rehabilitation protocols for patients with SCI. The development of personalized, AI-assisted approaches may contribute to improved functional outcomes and quality of life. However, realizing this potential requires rigorous validation in SCI-specific populations, addressing current technological limitations, and establishing appropriate regulatory and ethical frameworks. Future research should focus on generating high-quality evidence to support the integration of AI monitoring into standard SCI care pathways.
