# Supplementary figures and images for: A narrative review of AI monitoring in postoperative pain management and functional rehabilitation for spinal cord injury
Source: Front Neurol. 2026 Jul 9;17:1838329. doi: 10.3389/fneur.2026.1838329 (PMC13393461; doi:10.3389/fneur.2026.1838329)

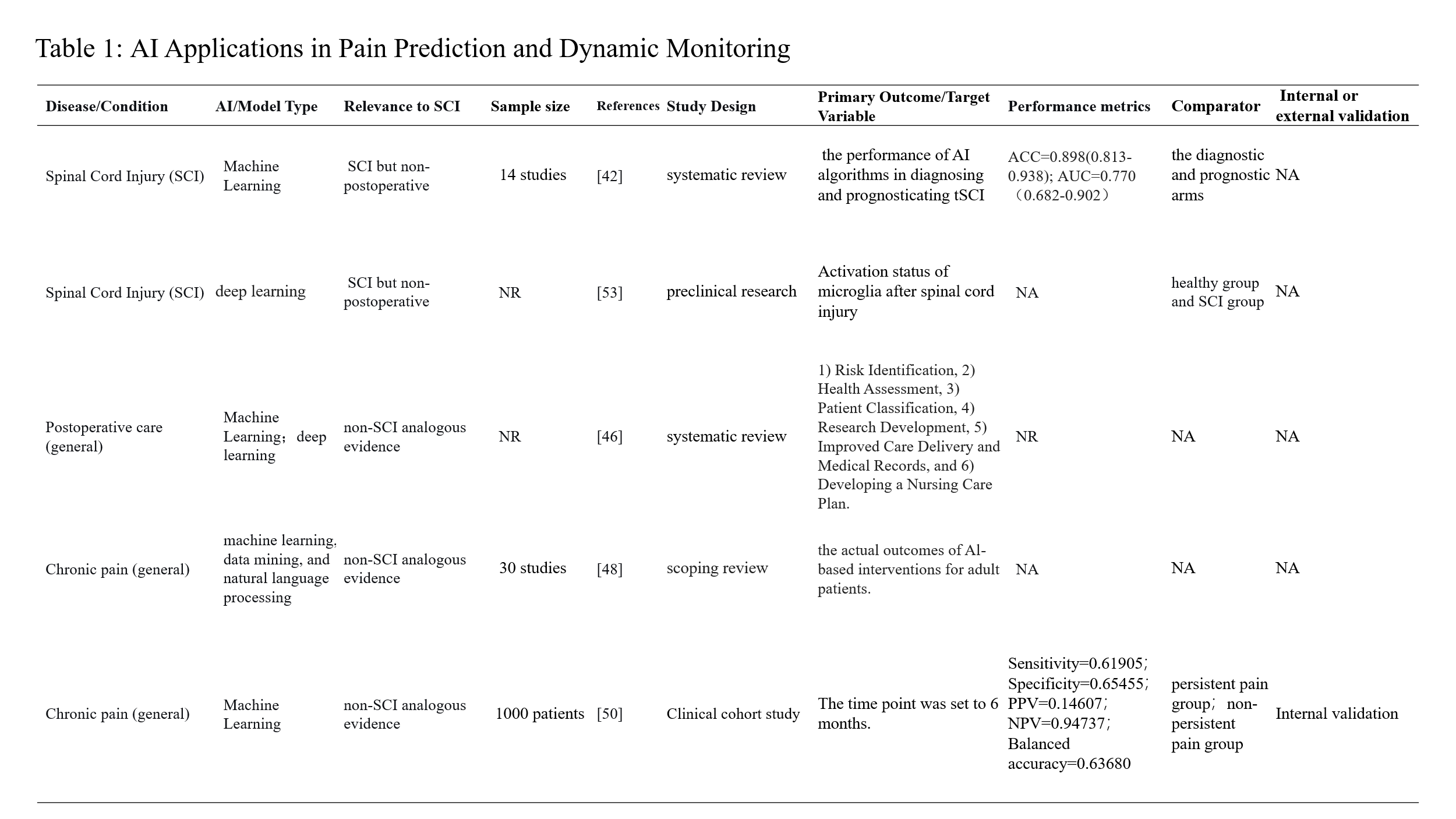

Supplement: Supplementary file 1 [file Table_1.docx]

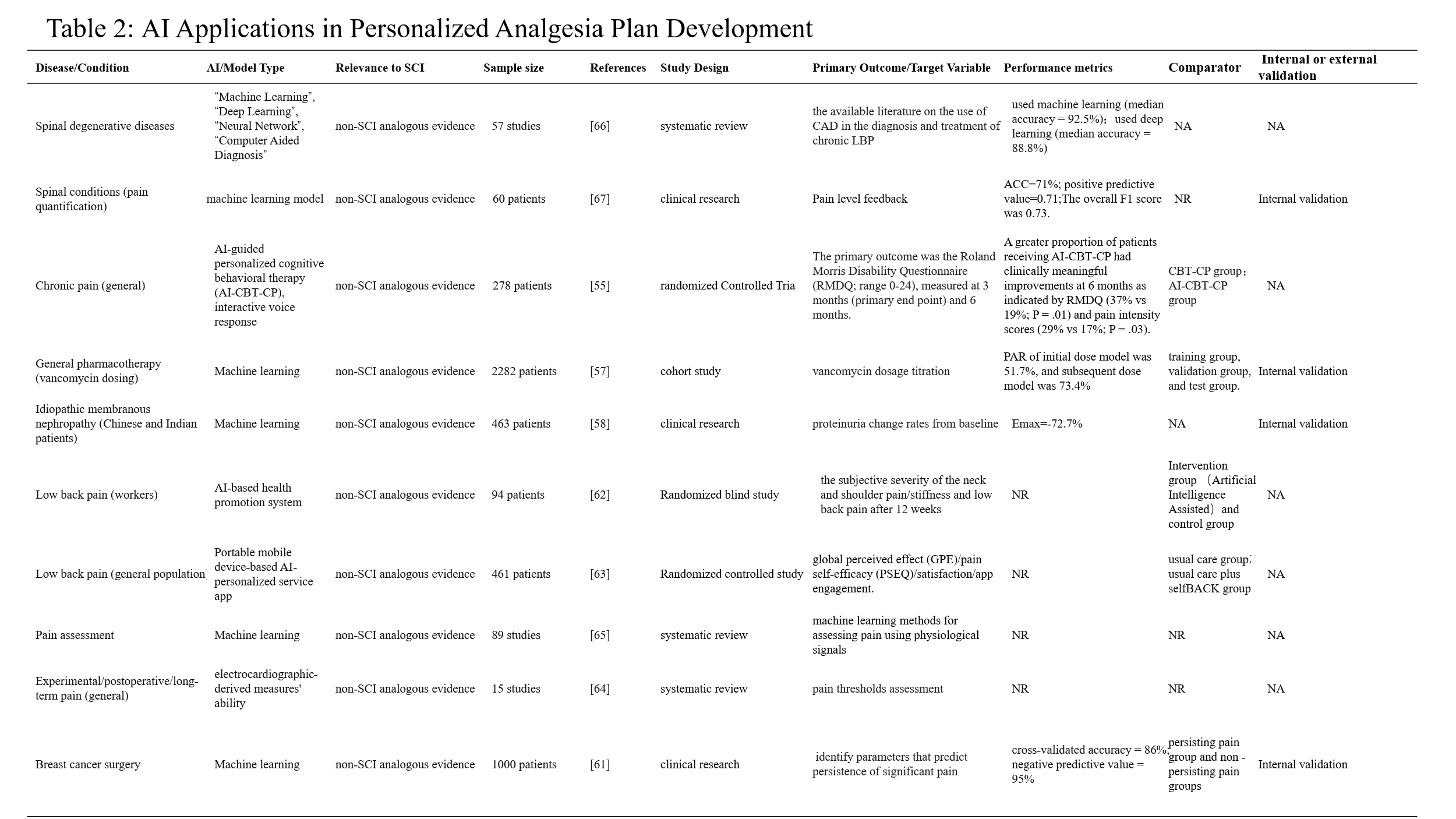

Supplement: Supplementary file 2 [file Table_2.docx]

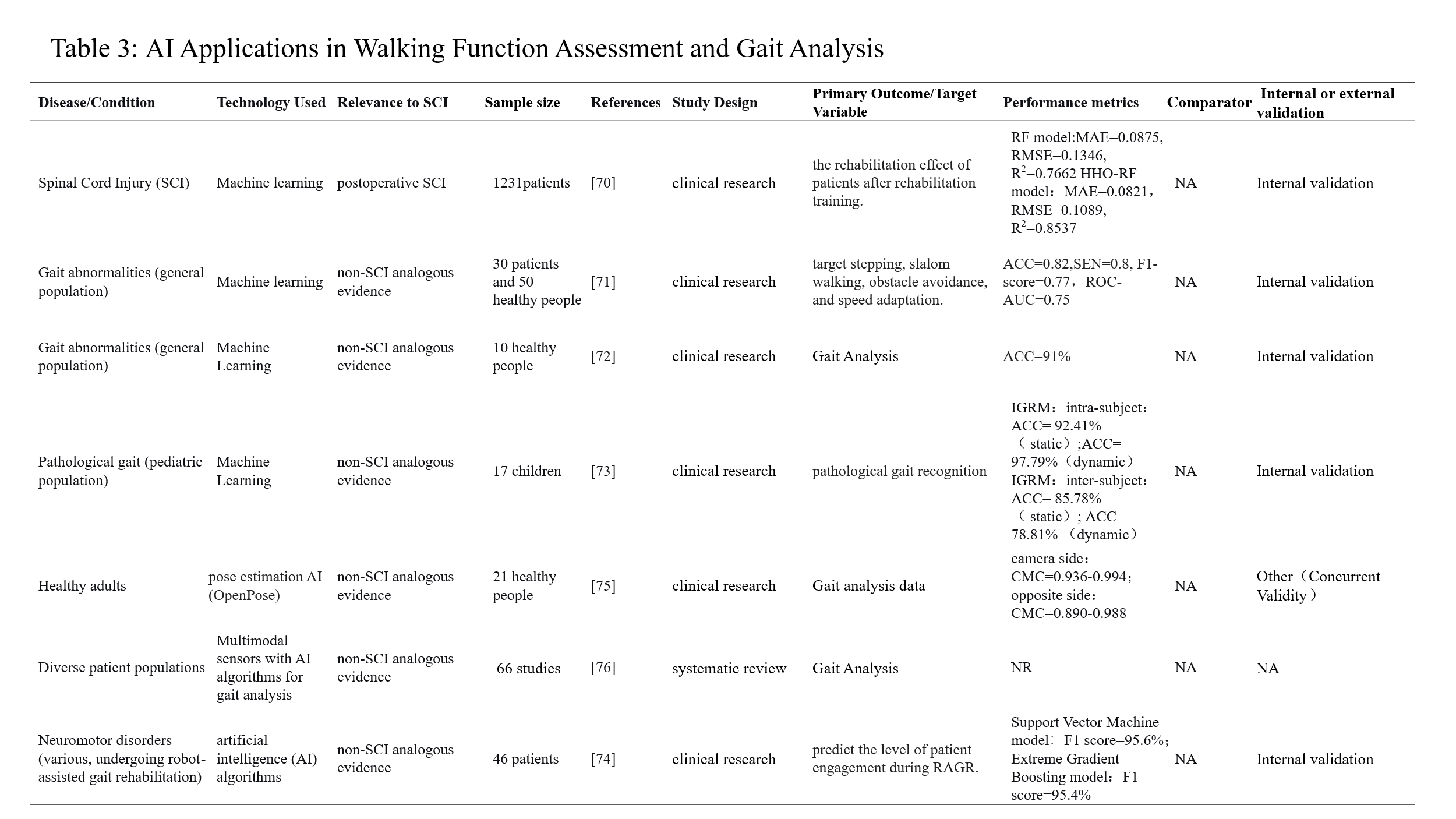

Supplement: Supplementary file 3 [file Table_3.docx]

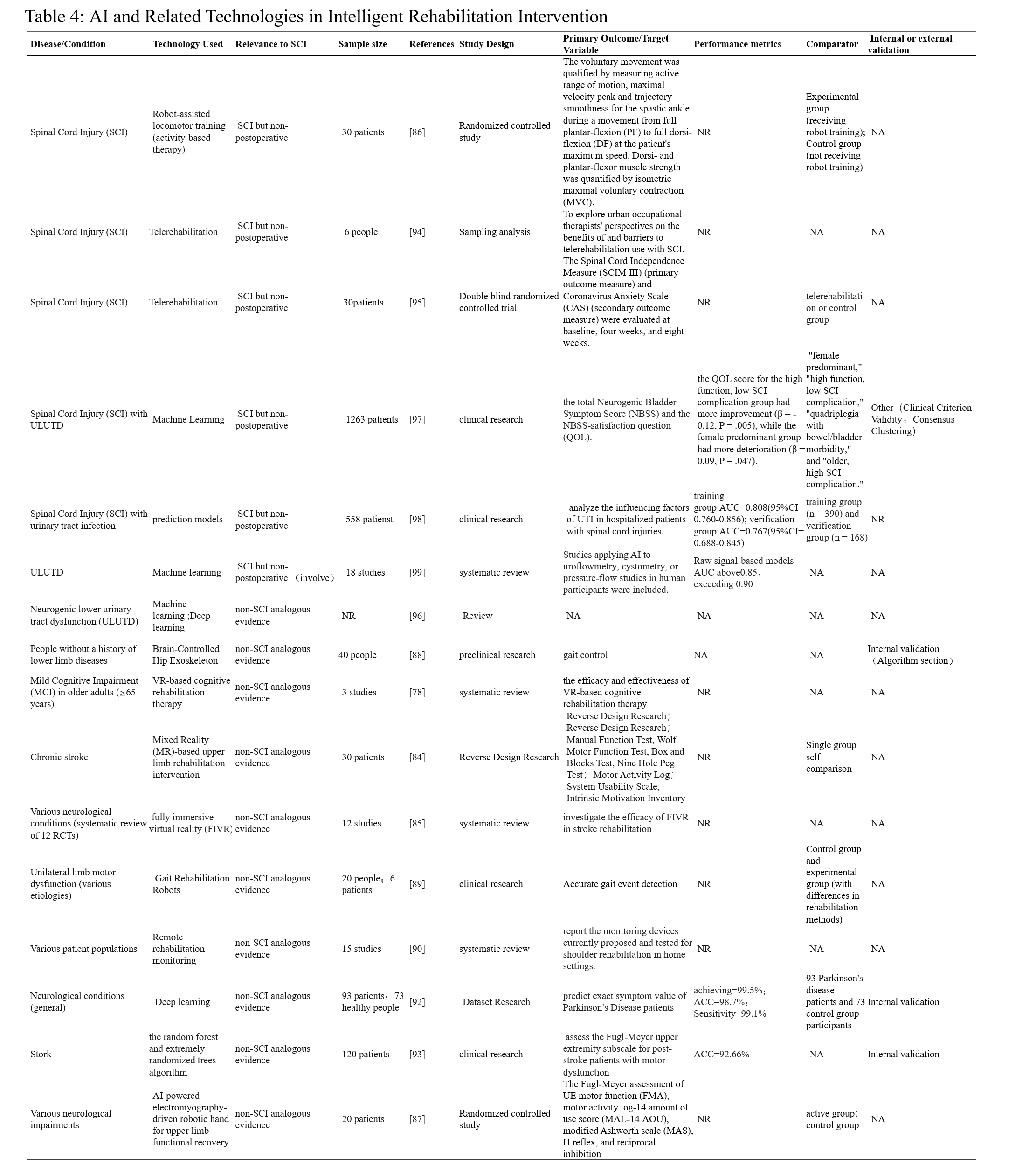

Supplement: Supplementary file 4 [file Table_4.docx]
