# Supplementary material for: A narrative review of AI monitoring in postoperative pain management and functional rehabilitation for spinal cord injury
Source: Front Neurol. 2026 Jul 9;17:1838329. doi: 10.3389/fneur.2026.1838329 (PMC13393461; doi:10.3389/fneur.2026.1838329)
Supplement: Supplementary file 5 [file Table_5.docx]

**Supplementary materials:**

Citation-to-claim audit（This audit primarily addresses references related to the main conclusions of this review; background or introductory references fall outside the scope of this audit.）

| **#** | **Claim in Manuscript** | **Reference** | **Supporting Text in Source (verbatim or precise paraphrase)** | **Match & Caveats in Manuscript** |
| --- | --- | --- | --- | --- |
| 1 | AI technology has been increasingly integrated into clinical settings, with AI-based monitoring systems demonstrating potential to enhance diagnostic accuracy, treatment efficiency, and patient experience. | [42] | AI demonstrates strong diagnostic accuracy (mean accuracy: 0.898) and moderate prognostic capability (mean AUC: 0.770) for tSCI. | Directly supported (review). |
| 2 | Furthermore, preliminary evidence suggests that AI-assisted monitoring may contribute to perioperative care. Studies indicate potential benefits in improving the success rate of spinal surgeries, reducing human error, optimizing perioperative workflows, and enhancing the quality of care. | [46] | It sheds light on potential avenues for future research, such as utilizing AI for the early identification of patients at high risk of readmission, patient classification, improving care delivery and medical records, and developing nursing care plans. | Indirectly supported (non-SCI analogous evidence).We added, "However, it should be noted that this finding is derived from a general surgical population and may not be directly generalizable to SCI patients without further validation.“ |
| 3 | However, it should be noted that this finding is derived from a general surgical population and may not be directly generalizable to SCI patients without further validation. | [47] | However, large, randomized trials documenting the benefit for patient improvements are still sparse. And the clinical uptake of explainable AI to improve implementation needs investigation. | Indirectly supported (non-SCI analogous evidence) |
| 4 | Personalized treatment strategies formulated through such approaches may hold promise for delivering more efficacious therapies to SCI patients. | [48] | Findings from this review suggest that using AI-based interventions has a positive effect on pain recognition, pain prediction, and pain self-management; however, most reports are only pilot studies. | Indirectly supported (non-SCI analogous evidence).We use 'may' to limit the conclusion. |
| 5 | Personalized treatment strategies formulated through such approaches may hold promise for delivering more efficacious therapies to SCI patients. | [49] | Results of these studies show promise for the improvement of patient outcomes. Current gaps in the research and subsequent directions for future study involve AI in optimizing and improving nerve stimulation and more thoroughly predicting patients' responses to treatment. | Indirectly supported (non-SCI analogous evidence).We use 'may' to limit the conclusion. |
| 6 | Personalized treatment strategies formulated through such approaches may hold promise for delivering more efficacious therapies to SCI patients. | [50] | Using a data-driven machine-learning approach, a short list of seven items from BDI and STAI is proposed as a basis for a predictive tool for the persistence of pain after breast cancer surgery. | Indirectly supported (non-SCI analogous evidence).We use 'may' to limit the conclusion. |
| 7 | Personalized treatment strategies formulated through such approaches may hold promise for delivering more efficacious therapies to SCI patients. | [51] | Our results indicate that simpler machine learning approaches might offer superior results; however, all of these techniques may play a promising role for developing smarter post-operative pain management strategies. | Indirectly supported (non-SCI analogous evidence).We use 'may' to limit the conclusion. |
| 8 | Personalized treatment strategies formulated through such approaches may hold promise for delivering more efficacious therapies to SCI patients. | [52] | However, realizing the full potential of AI in SCI care requires ongoing research, interdisciplinary collaboration, and the development of comprehensive datasets. As AI continues to evolve, it is expected to play an increasingly vital role in enhancing the outcomes of patients with SCI. | Directly supported (review). |
| 9 | Personalized treatment strategies formulated through such approaches may hold promise for delivering more efficacious therapies to SCI patients. | [53] | This robust computational technique has potential for analyzing microglial activation across various neuropathologies and demonstrating the usefulness of nanovectors in modifying microglia in SCI and other neurological disorders. It has the ability to speed development in this crucial sector by providing a standardized and objective way to compare therapeutic options. | Directly supported (preclinical research). |
| 10 | Although this study focused on general chronic pain rather than SCI specifically, its findings suggest that AI-assisted psychological interventions warrant investigation in SCI populations. | [55] | The findings of this randomized comparative effectiveness trial indicated that AI-CBT-CP was noninferior to therapist-delivered telephone CBT-CP and required substantially less therapist time. Interventions like AI-CBT-CP could allow many more patients to be served effectively by CBT-CP programs using the same number of therapists. | Indirectly supported (non-SCI analogous evidence).We use 'its findings suggest that AI-assisted psychological interventions warrant investigation in SCI populations.' to limit the conclusion. |
| 11 | While direct evidence in SCI pain management is limited, studies in other clinical contexts demonstrate the potential of this approach. | [56] | These applications provide an opportunity for ML and pharmacometrics to operate in an integrated manner to provide clinical decision support for precision dosing. | Indirectly supported (non-SCI analogous evidence).We use 'While direct evidence in SCI pain management is limited, studies in other clinical contexts demonstrate the potential of this approach.' to limit the conclusion. |
| 12 | These examples illustrate the broader applicability of AI-driven dosage optimization, which could potentially be adapted for analgesic management in SCI, though direct studies are needed. | [57] | Our results show that the new AI-assisted dosage titration approach has the potential to improve the traditional approaches. This is especially useful to guide decision making for inexperienced doctors in making consistent and safe dosing recommendations for high-risk medications like vancomycin. | Indirectly supported (non-SCI analogous evidence).We use 'These examples illustrate the broader applicability of AI-driven dosage optimization, which could potentially be adapted for analgesic management in SCI, though direct studies are needed.' to limit the conclusion. |
| 13 | These examples illustrate the broader applicability of AI-driven dosage optimization, which could potentially be adapted for analgesic management in SCI, though direct studies are needed. | [58] | The present study is the first time to establish a machine learning model for predicting effects of tacrolimus on pro teinuria in Chinese and Indian patients with IMN and for achieving better therapeutic effects of tacrolimus on protein uria in patients with IMN, tacrolimus concentration range need to be maintained at 4–10 ng/ml for at least 1.72 months. | Indirectly supported (non-SCI analogous evidence).We use 'These examples illustrate the broader applicability of AI-driven dosage optimization, which could potentially be adapted for analgesic management in SCI, though direct studies are needed.' to limit the conclusion. |
| 14 | Given the challenges of opioid management in SCI, this application may offer a useful monitoring strategy; however, its feasibility and performance in the postoperative SCI setting have not been directly studied. | [60] | The novel prototype uses innovative AI-based techniques to automate searching for, extracting, and analyzing clinically useful information captured in unstructured text in EHRs. It increases efficiency in harnessing real-world data for opioid drug safety and increases the usability of the data to support regulatory review while decreasing the manual research burden. | Indirectly supported (non-SCI analogous evidence).We use 'however, its feasibility and performance in the postoperative SCI setting have not been directly studied.' to limit the conclusion. |
| 15 | While this population differs from SCI patients, the methodological approach—using machine learning to integrate multiple clinical parameters for pain prediction—could inform future research in SCI. | [61] | The present machine-learned analysis showed that, even with a large set of parameters acquired from a large cohort, early identification of these patients is only partly successful. This indicates that more parameters are needed for accurate prediction of persisting pain. | Indirectly supported (non-SCI analogous evidence).We use 'could inform future research in SCI' to limit the conclusion. |
| 16 | These findings in low back pain populations suggest that similar approaches might be explored for SCI-related pain, though the underlying pain mechanisms differ substantially. | [62] | This study shows that the short exercises provided by the AI-assisted health program improved both neck/shoulder pain/stiffness and low back pain in 12 weeks. Further studies are needed to identify the elements contributing to the successful outcome of the AI-assisted health program. | Indirectly supported (non-SCI analogous evidence).We use 'might be explored for SCI-related pain, though the underlying pain mechanisms differ substantially.' to limit the conclusion. |
| 17 | These findings in low back pain populations suggest that similar approaches might be explored for SCI-related pain, though the underlying pain mechanisms differ substantially. | [63] | selfBACK offers an opportunity to support people with LBP and provides clinicians with an additional tool for their patients, even those with depression or high levels of stress. This highlights the potential for digital health interventions for chronic pain. | Indirectly supported (non-SCI analogous evidence).We use 'might be explored for SCI-related pain, though the underlying pain mechanisms differ substantially.' to limit the conclusion. |
| 18 | This physiological approach may have more applicability to SCI, as autonomic dysfunction is common in this population. | [64] | This systematic review investigated electrocardiographic-derived autonomic tone and found that increased parasympathetic tone could predict pain reduction in different types of pain. | Indirectly supported (non-SCI analogous evidence).We use 'This physiological approach may have more applicability to SCI.' to limit the conclusion. |
| 19 | These applications may relevant to SCI care, as many SCI patients have concomitant spinal pathology. | [65] | It examined the current advances in machine learning approaches for the identification and measurement of pain using physiological signals. The review intended to highlight the currently available objective methods of acute and chronic pain assessment and the need to substantiate a method with better sensitivity and validity for clinical and research purposes. | Indirectly supported (non-SCI analogous evidence).We use 'These applications may relevant to SCI care, as many SCI patients have concomitant spinal pathology.' to limit the conclusion. |
| 20 | These applications may relevant to SCI care, as many SCI patients have concomitant spinal pathology. | [66] | The best performing systems were developed to diagnose degenerative changes of the spine from imaging data, with average accuracy rates >80%. However, notable outcomes were also reported for CAD tools executing different tasks including analysis of clinical, biomechanical, electrophysiological, and functional imaging data. Further studies are needed to better define the role of CAD in LBP care. | Indirectly supported (non-SCI analogous evidence).We use 'These applications may relevant to SCI care, as many SCI patients have concomitant spinal pathology.' to limit the conclusion. |
| 21 | These applications may relevant to SCI care, as many SCI patients have concomitant spinal pathology. | [67] | Herein, we propose a machine learning–based methodology to quantify pain using speech data from smartphones and self-reported pain surveys in a cohort of patients with spine disease. Using our prediction model as a baseline, future models can improve on our framework to better evaluate pain levels in patients with spine disease. | Indirectly supported (non-SCI analogous evidence).We use 'These applications may relevant to SCI care, as many SCI patients have concomitant spinal pathology.' to limit the conclusion. |
| 22 | This evidence suggests that the application of artificial intelligence has potential positive implications for the functional recovery of SCI patients after surgery. | [70] | In clinical treatment, HHO-RF can accurately predict the discharged ADL score and provide a reasonable direction for patients to choose rehabilitation programs. | Directly supported (clinical research). |
| 23 | While these findings demonstrate the technical feasibility of AI-based gait analysis, it should be noted that these studies were conducted in general populations with gait abnormalities rather than specifically in SCI patients. The transferability of these algorithms to SCI-specific gait patterns requires further validation. | [71] | The AdaCost identifies individuals with GAD and facilitates clinical decision-making. This advances the future development of user-friendly interfaces and computer-aided diagnosis systems. | Indirectly supported (non-SCI analogous evidence).We use 'The transferability of these algorithms to SCI-specific gait patterns requires further validation.' to limit the conclusion. |
| 24 | While these findings demonstrate the technical feasibility of AI-based gait analysis, it should be noted that these studies were conducted in general populations with gait abnormalities rather than specifically in SCI patients. The transferability of these algorithms to SCI-specific gait patterns requires further validation. | [72] | The IMU-based system shows promise for accurate gait assessment in patients with joint impairments, suggesting future research for clinical application improvements in rehabilitation and patient management. | Indirectly supported (non-SCI analogous evidence).We use 'The transferability of these algorithms to SCI-specific gait patterns requires further validation.' to limit the conclusion. |
| 25 | While these findings demonstrate the technical feasibility of AI-based gait analysis, it should be noted that these studies were conducted in general populations with gait abnormalities rather than specifically in SCI patients. The transferability of these algorithms to SCI-specific gait patterns requires further validation. | [73] | The experimental results reveal the potential for the computer supervision of non-pathological and pathological gaits in the plantar-pressure patterns of children and for providing feedback in the application of gait-abnormality rectification. | Indirectly supported (non-SCI analogous evidence).We use 'The transferability of these algorithms to SCI-specific gait patterns requires further validation.' to limit the conclusion. |
| 26 | This study included patients with various neuromotor disorders, and although SCI was not the sole focus, the methodological approach may inform future research in SCI rehabilitation. | [74] | The study displayed the effectiveness of psychophysiology-based AI models in predicting rehabilitation engagement, thus promoting their practical application for personalized care and improved clinical health outcomes. | Indirectly supported (non-SCI analogous evidence).We use 'although SCI was not the sole focus, the methodological approach may inform future research in SCI rehabilitation.' to limit the conclusion. |
| 27 | However, this validation was performed in healthy adults rather than SCI patients, and further studies are needed to establish its accuracy in SCI populations with varying levels of impairment. | [75] | This study aimed to clarify objective measures for absolute error and waveform pattern similarity in gait analysis using pose estimation AI (OpenPose). Additionally, we investigated the feasibility of simultaneous measuring both lower limbs using a single camera from one side. | Indirectly supported (non-SCI analogous evidence).We use 'However, this validation was performed in healthy adults rather than SCI patients, and further studies are needed to establish its accuracy in SCI populations with varying levels of impairment.' to limit the conclusion. |
| 28 | This systematic review provides a comprehensive overview of the field, but it is important to note that the included studies covered diverse patient populations, and the specific benefits for SCI gait analysis remain to be fully characterized. | [76] | The findings of this review suggest that a smart, portable, wearable-based gait and balance assessment system can be developed using multimodal sensing of the most cutting-edge, clinically relevant tools and technology available. The information presented in this article may serve as a vital springboard for such development. | Indirectly supported (non-SCI analogous evidence).We use 'but it is important to note that the included studies covered diverse patient populations, and the specific benefits for SCI gait analysis remain to be fully characterized.' to limit the conclusion. |
| 29 | While this evidence supports VR efficacy in MCI, its direct applicability to SCI rehabilitation requires investigation, as the mechanisms of cognitive impairment and rehabilitation needs differ between these populations. | [78] | Nevertheless, although scarce, results of the present review suggest that VR-CRT may be paramount in treating MCI for its additional ecological and adaptive advantages, as all of the studies highlighted that it was at least as effective as conventional CRT for all the outcome measures. | Indirectly supported (non-SCI analogous evidence).We use 'its direct applicability to SCI rehabilitation requires investigation, as the mechanisms of cognitive impairment and rehabilitation needs differ between these populations.' to limit the conclusion. |
| 30 | However, these reviews included studies across various neurological conditions, and the specific evidence for SCI remains limited. | [84] | Our results support the clinical effectiveness of mixed reality interventions that satisfy the motor learning principles for upper limb rehabilitation in chronic stroke survivors. This characteristic, together with the low cost of the system, its portability, and its acceptance could promote the integration of these systems in the clinical practice as an alternative to more expensive systems, such as robotic instruments. | Indirectly supported (non-SCI analogous evidence).We use 'and the specific evidence for SCI remains limited.' to limit the conclusion. |
| 31 | However, these reviews included studies across various neurological conditions, and the specific evidence for SCI remains limited. | [85] | Therefore, FIVR represents an adaptable, multi-faceted rehabilitation tool that can be considered in post-stroke rehabilitation, improving the compliance of the patients to the treatment and increasing the level of functioning and quality of life of stroke survivors. | Indirectly supported (non-SCI analogous evidence).We use 'and the specific evidence for SCI remains limited.' to limit the conclusion. |
| 32 | These studies provide direct evidence in SCI populations, supporting the potential of robot-assisted interventions. | [86] | The improvements of the kinematic and kinetic parameters of the ankle voluntary movement, and their correlation with the functional assessments, support the therapeutic effect of robotic-assisted locomotor training on motor impairment in chronic iSCI. | Directly supported (Randomized controlled study). |
| 33 | While this study included patients with various neurological impairments, the findings suggest that AI-integrated rehabilitation robots may have applications in SCI upper limb rehabilitation. | [87] | The AI-integrated EMG-driven robot improved UE motor function  and spasticity, which persisted for 4 weeks. This robot hand might be useful for UE rehabilitation of patients with stroke. | Indirectly supported (non-SCI analogous evidence).We use 'the findings suggest that AI-integrated rehabilitation robots may have applications in SCI upper limb rehabilitation.' to limit the conclusion. |
| 34 | This represents a promising proof-of-concept study that could be conducted in patients with spinal cord injury (SCI), demonstrating the feasibility of brain-computer interface-controlled exoskeletons for lower-limb rehabilitation. | [88] | These findings validate the system’s efficacy in enabling efficient and stable human–exoskeleton collaboration. The proposed framework offers valuable insights for future development of multi-joint exoskeleton control systems. Future studies will focus on clinical translation, with validation of system safety, usability, and therapeutic efficacy in patients with hip motor impairments, which is essential for optimizing the system for diverse rehabilitation needs. | Indirectly supported (non-SCI analogous evidence).We use 'This represents a promising proof-of-concept study that could be conducted in patients with spinal cord injury (SCI)' to limit the conclusion. |
| 35 | This study's population included patients with various etiologies of unilateral dysfunction, limiting conclusions specific to SCI. | [89] | Compared to the conventional thresholding method, this work presents a simple and robust application for gait event detection in healthy and hemiplegic subjects by one inertial sensor. The linear regression model can be applicable to different subjects walking at various stride | Indirectly supported (non-SCI analogous evidence).We use 'This study's population included patients with various etiologies of unilateral dysfunction, limiting conclusions specific to SCI.' to limit the conclusion. |
| 36 | However, this study's methodology and validation population require careful examination before extrapolation to SCI. | [92] | This is the first study to use a deep learning regression approach to predict exact symptom value of Parkinson’s Disease patients. Results show that this approach can be effectively employed as a disease severity assessment tool using wearable sensors. | Indirectly supported (non-SCI analogous evidence).We use 'However, this study's methodology and validation population require careful examination before extrapolation to SCI.' to limit the conclusion. |
| 37 | This stroke-specific tool demonstrates the feasibility of AI-based remote monitoring for neurological rehabilitation, suggesting similar approaches could be developed for SCI. | [93] | The application of the proposed method enables precision assessment of patients' upper extremity motor function, thereby facilitating more personalized rehabilitation programs to achieve optimal recovery outcomes. | Indirectly supported (non-SCI analogous evidence).We use 'This stroke-specific tool demonstrates the feasibility of AI-based remote monitoring for neurological rehabilitation, suggesting similar approaches could be developed for SCI.' to limit the conclusion. |
| 38 | These patient-reported outcomes highlight the perceived value of remote rehabilitation among individuals with SCI. | [94] | As practitioner concerns are identified and addressed, telerehabilitation may increase in the U.S. health care system, potentially facilitating an alternative treatment delivery method for underserved | Supported； SCI but non-postoperative (Sampling analysis). |
| 39 | These patient-reported outcomes highlight the perceived value of remote rehabilitation among individuals with SCI. | [95] | Telerehabilitation intervention is safe, feasible, and effective in improving self-care and mobility domains in persons with spinal cord injuries during the pandemic. It is also effective in reducing the anxiety related to the coronavirus in this population. | Supported； SCI but non-postoperative (Double blind randomized controlled trial). |
| 40 | These advances suggest that AI-based monitoring could extend beyond pain management and motor rehabilitation to address NLUTD, a critical yet often undermanaged domain in SCI care. However, most studies remain limited by small sample sizes and lack of external validation, underscoring the need for large-scale, multicenter validation in SCI-specific populations before clinical implementation. | [96] | Automated systems now enable precise interpretation of complex bladder signals, multimodal data integration, and real-time prediction of treatment outcomes, marking a shift toward data-driven precision medicine. Nevertheless, most published studies remain limited by small, single-center datasets and a lack of external validation. Broader clinical adoption will require multicenter collaboration, adherence to standardized reporting frameworks such as TRIPOD-ML and PROBAST-AI, and integration of explainable AI to ensure transparency, reproducibility, and clinician trust. | Indirectly supported (non-SCI analogous evidence).We use 'However, most studies remain limited by small sample sizes and lack of external validation, underscoring the need for large-scale, multicenter validation in SCI-specific populations before clinical implementation.' to limit the conclusion. |
| 41 | These advances suggest that AI-based monitoring could extend beyond pain management and motor rehabilitation to address NLUTD, a critical yet often undermanaged domain in SCI care. However, most studies remain limited by small sample sizes and lack of external validation, underscoring the need for large-scale, multicenter validation in SCI-specific populations before clinical implementation. | [97] | This study demonstrates the utility of machine learning in uncovering bladder-relevant phenotypes among SCI patients. Future research should explore cluster-based targeted strategies to enhance bladder-related outcomes and QOL in SCI. | Directly supported (clinical research). |
| 42 | These advances suggest that AI-based monitoring could extend beyond pain management and motor rehabilitation to address NLUTD, a critical yet often undermanaged domain in SCI care. However, most studies remain limited by small sample sizes and lack of external validation, underscoring the need for large-scale, multicenter validation in SCI-specific populations before clinical implementation. | [98] | The nomogram prediction model based on the above factors can simply and effectively predict the risk of UTI in hospitalized patients with spinal cord injury, which is helpful for clinical medical staff to identify high-risk groups early and implement prevention, treatment, and nursing strategies in time. | Directly supported (clinical research). |
| 43 | These advances suggest that AI-based monitoring could extend beyond pain management and motor rehabilitation to address NLUTD, a critical yet often undermanaged domain in SCI care. However, most studies remain limited by small sample sizes and lack of external validation, underscoring the need for large-scale, multicenter validation in SCI-specific populations before clinical implementation. | [99] | At present, AI-assisted urodynamic interpretation should be considered exploratory, and robust prospective studies with independent external validation are required before routine clinical implementation. | Supported； SCI but non-postoperative (systematic review). |
